# Dr. Rentoul's Rhapsody

**Published:** 1895-07-06

**Authors:** 


### Dr. Rentoul's Rhapsody.

Every measure brings forth its man, and the Midwives Registration Bill has produced Dr. Rentoul, the champion of the lower class of medical practitioners, a man whose words truly are many but whose ideas are few. He in turn has produced a pamphlet which we have read with just this amount of interest—that we looked for, in it, the worst that could possibly be said against the Bill. In the way of abuse we were in no way disappointed. He calls it on his title-page " a disease-producing and death-dealing proposal: an insult to poor women," and straightway on the next page adds to his adjectives the terms "crime-producing," " degrading," and " dangerous," and goes on to say, " It would seem as if a few medical practitioners were possessed by some deep hatred towards pregnant women and infants, and were determined to endanger their lives to the greatest extent." These are terms which no man with any sense of responsibility has any right to use unless he can show that the Bill will do some fresh and hitherto unknown injury to the women of England and introduce some new peril into their time of tribulation. Yet what do we find? A dissertation largely devoted to emphasising the perils of child-birth and the dangers arising from the employment of ignorant midwives, with all of which we quite agree, and ending most illogically by pouring abuse on the only reasonable attempt which has been made to improve this dangerous condition of affairs. We do not pretend that the Bill as it stands is perfect; we may even doubt whether it will fully attain the purpose for which it has been brought forward, viz., the protection of the poorer class of women from the tender mercies of ignorant midwives; but there surely can be no doubt about the general principle that if midwives are to continue attending confinements they should be educated and supervised in their art. Our chief objection is that the Bill does not go far enough. The Bill proposes to educate midwives. We would go further, and not allow them to practise unless they were so educated. That is our sole difference with the promoters of the Bill. We are not concerned either for the doctors or the midwives, we merely ask that the subject shall not be trifled with, and that the Bill shall give protection to the women of England.

What Dr. Rentoul asks, however, is quite another thing. He is careful not to be too definite. His pamphlet is a furious attack upon the Bill; but he avoids laying down any scheme by which the present evils, which he admits, may be prevented in the future. Admitting the evils—the deaths, the diseases, the still-births—which take place under the present system, he closes his book with the following inane proposition: "The present provisions made for poor pregnant women are amply sufficient." Are there not, he says, sufficient medical practitioners to attend all confinements? The difference between Dr. Rentoul and ourselves is radical. We would mend the Bill for the more complete protection of the women; he would throw it out—for the protection, apparently, of the medical profession. The great mistake made by Dr. Rentoul and all his following is that they shut their eyes to the fact that at the present time midwifery is perfectly legally practised in all parts of England by absolutely ignorant women. To refuse legislation is to sanction the continuance of this evil custom with all its dangers; it would in no way help forward that millennium when all women will be attended by fifth-year medical students and by qualified medical practitioners who, according to Dr. Rentoul, "are willing—nay, anxious—to attend a confinement for a fee varying from 5s. to 10s." A certain set of medical men seem to think that the world can get on very well without midwives, although what is to happen in the scores and hundreds of little villages miles from any doctor, we do not know; any more than we pretend to know the sort of town doctor who will lay himself out for 5s. midwifery. In the meantime the midwives exist, and if they cannot be ended it is a scandal that they should not be mended, and that right quickly. Dr. Rentoul's attitude on this question is dishonouring to the medical profession, impracticable, unstatesmanlike, and in the highest sense unworthy of the best traditions of medical science. His lucubrations are calculated to emphasise the importance of speedy legislation for midwives and to bring the medical profession into contempt. He should be smartly rebuked and repudiated by his confrères.

